# Supporting siblings during the critical illness hospitalization of a child: learning from experience

**DOI:** 10.3389/fped.2024.1337491

**Published:** 2024-08-26

**Authors:** Karen Dryden-Palmer, Alexis Shinewald, Kimberly O'Leary

**Affiliations:** ^1^ Paediatric Intensive Care Unit, The Hospital for Sick Children, Toronto, ON, Canada; ^2^ Child Health Evaluative Sciences, The Hospital for Sick Children, Toronto, ON, Canada; ^3^ Child Life Program, The Hospital for Sick Children, Toronto, ON, Canada

**Keywords:** pediatric, child, critical illness, intensive care, child and family-centered care, siblings

## Abstract

**Introduction:**

Childhood critical illness impacts the entire family of the critically ill patient. Disruptions to usual family rhythms and routines, established relationships, physical relocations or shifts in caregivers, and the uncertainty about the patient's well-being can have significant impacts on siblings and other connected children in the family. Promoting and facilitating family interactions and engaging younger family members in the hospital experience have been shown to reduce patient and family anxiety, enhance family adaptation, and improve child and family outcomes. The critical care team can implement evidence-informed approaches to address and mitigate challenges for families and provide developmentally aligned support to impacted siblings.

**Aim:**

This conceptual paper describes the potential impacts of a critical illness hospitalization on siblings, approaches to supporting siblings, and practical interventions drawn from a synthesis of the current literature and the author's practice experience caring for critically ill children and their families.

**Data sources:**

A traditional review and narrative analysis moderated by the authors and supported by lived experience.

**Conclusions:**

There is a range of impacts of a critical illness hospitalization on siblings and young family members of the patient. Providing consistent, transparent, and supportive child, sibling, and whole family-centered care can improve the experience and outcomes for the child and family.

## Introduction

Childhood critical illness affects the entire family unit. This disruption has significant impacts on siblings and other children in the family due to changes in family rhythms and routines, physical relocation, changes in caregivers, and uncertainty of the patient's well-being (1–3). The term “sibling” acknowledges all the children and youth who are part of the patient's relationship systems regardless of their biological or relational connections. All children and youth members of the patient's family or immediate community may benefit from these recommendations (2).

Approaches and interventions to support family members during the critical care experience have evolved to include family presence, emotional care for family members, shared decision-making, and participatory care planning. Parents can contribute to their child's care through multiple avenues including participation in medical rounding and providing direct care to the child (1). Less attention has been paid to integrating siblings of the ill child. The PICU environment and processes are designed for adults. Young family members' needs may not be easily identified and thus siblings may be poorly integrated into the plan of care (4).

Developing systems and approaches to care that promote and nurture family interactions and engage younger family members can have a positive impact on the patient and family (5–7). Sibling-inclusive practices can reduce anxiety for the sibling and parents, enhance family adaptation, and shorten recovery time (8). When the integrity of the whole family is preserved, patients and their families are more satisfied with their care, achieve growth, and may develop skills applicable to future family challenges. In this conceptual paper, we discuss the potential impacts of childhood critical illness hospitalization on siblings. We describe approaches and practical interventions, drawn from the literature and practice experience, that facilitate care for critically ill children, siblings and families.

## Impacts of critical hospitalization of a child on siblings

Well siblings of critically ill patients are exposed to many stresses. These stresses may be similar to those experienced by parents and adult family members. For example, they worry about the ill child and experience fear of the unknown. Children also experience challenges unique to being a sibling, for example, disruption to their daily activities and anxiety related to separation from family members and caregivers (1). Siblings can be displaced from familiar environments, their home, school, or community as their families rearrange childcare and home responsibilities. It is common for siblings to feel confusion, jealousy, resentment, and guilt or to blame themselves for the distress they witness (8, 9, 10). Siblings may also experience isolation and feel forgotten by the family, have limited access to their usual coping mechanisms, and may not have the ability to ground their interpretation of events (1, 3). Siblings may create fictional causes or reasons for the illness and project unrealistic or inaccurate understandings about the patient's situation and the resulting disruption to the family (1).

The adults who support siblings can also face pragmatic, emotional, and interpersonal challenges. Parents and family caregivers may experience conflict related to their role and obligations to the patient (11, 12). Families report struggling with separation during hospitalization and experience tension between the needs of the patient and those of other children in the family (13).

Environmental and situational stressors are numerous as well. Parents must adapt to the intensive care environment, deal with overwhelming informational needs, and are required to function in a novel and unanticipated context (14). Parents require energy, support, and resources to navigate the unpredictable and dynamic elements of their child's critical illness (2).

Parents may also feel uncertain about how to best support the well child. How to be a parent in a critical illness situation may not be intuitively understood, and the skills required to do so effectively may be unfamiliar or lacking. Families report a desire to protect their well children at home while also feeling the need to inform and engage them (15). Thus, parents may need guidance and support to address the needs of the well children in the family.

Family perspectives during critical illness are shaped by the context of the child's hospitalization. The individual sociocultural character of the family, along with their goals, expectations, and preferred roles, shapes the needs of the family and its members including siblings (16). By acknowledging these tensions, anticipating needs, and providing validation, the critical care interprofessional team can assist each family to successfully navigate these challenges and ensure all members including siblings are supported.

## Principles guiding sibling support

The guiding principles of family-centered care (FCC)—respect and dignity, information sharing, participation, and collaboration—inform approaches to ensuring sibling well-being (17). Interventions addressing sibling needs are therefore strength-based, aligned with principles of FCC, and are intentionally fit to the child and family context while remaining sensitive to the clinical situation. These foundational principles were drawn from an informal traditional search of the current evidence supported by a medical librarian. Selected publications were reviewed by all authors, and principles were developed through consensus.

Sibling support and the initiation of a sibling care plan aligned with these principles can be provided by any knowledgeable members of the healthcare team including social workers, child life specialists, nurses, physicians, developmental specialists, and family experience navigators. Table 1 illustrates the six principles guiding sibling care and the associated objectives for critical care providers.

**Table 1:** Principles guiding sibling support in critical care.

Principle	Factors	Objective
Family as context	Family as defined by family (18)	Sibling support offered reflects the family's values, priorities, experience, understanding, tone, and preferences
	Family culture, roles, and expectations are unique to each family (19)	
Strength-based	Families bring collective and individual strengths to the experience of critical illness hospitalization (20)	Approaches to sibling support acknowledge and build on the existing strengths of each individual and the family
Individualized	The needs of siblings will evolve throughout the experience (21)	Approaches and interventions for sibling support are specific to the individual, developmentally aligned, and fit the unique needs of each sibling
	Sibling age and stage impact needs (9)	
Predictive and responsive	Family responses and needs vary and may be dynamic throughout the experience (10)	Sibling support anticipates predictable elements of the experience and responds to changes and unexpected situations
	Family and individual resources and capacity will vary through the course of the experience (22)	Ongoing assessment and adaptation of the plan for sibling support are performed
Fit to clinical situation	Critical illness experiences vary in their trajectory, uncertainty, risks, and outcomes	Sibling care is flexible and adapted to fit the current and predicted contexts of the clinical situation
	Goals of care and priorities can shift quickly or evolve over time	
Ongoing and consistent	Transparent reciprocal information sharing is essential (10)	An individual or small number of consistent providers with dedicated time work to develop a trusting rapport with the sibling
	Opportunities to clarify and build cumulative understandings are important across the critical illness experience (21)	Continuity of therapeutic relationship and approach is provided for siblings across the critical care experience

## Goals for sibling support

There are several interrelated goals for addressing sibling needs during a child's critical hospitalization. Achieving these goals will mutually benefit the sibling, the family, and the patient. Sibling support aims to (1) facilitate sibling engagement through active exploration and validation of needs, (2) acknowledge and honor relationships, (3) help the sibling to help the patient, and (4) normalize the sibling's responses and the critical care experience. Table 2 illustrates these goals, associated considerations, and examples of potential interventions for each.

**Table 2:** Goals and considerations for sibling support activities.

Goal	Purpose	Considerations	Actions/interventions
Proactive engagement and empowerment of sibling	Create safety and build rapport	Cognitive abilities, social and emotional development of both the patient and the sibling(s)	Assess the sibling's developmental level and needs
	Integrate siblings into the patient's and family's experience	Individual preferences and strengths	Engage and educate parents and family members as appropriate
	Facilitate family support of siblings	Family culture, norms, and expectations	Uncover and address misconceptions and fears about the cause/meaning of the illness
			Match explanations to the child's understanding and family's preferences (language, pace, and tone)
			Support expressive play and intentional expression, for example, art/journaling
			Establish consistent individual(s) when possible and support continuity of plan of support throughout the critical care interval
			Facilitate intentional integration of sibling input, for example, selecting items from the home to be kept at the bedside, decorating the patient's space, and choosing games or tasks when visiting
Acknowledge and honor relationships	Create opportunities for safe expression of emotion	Sibling and patient's capacity to participate in relational interventions	Facilitate interactions and bonding opportunities between siblings (see Supplementary Table S1 for examples)
	Maintain and strengthen family connections	Engage the whole family in interventions	Provide learning materials to support discussion about the patient’s experience, for example, books, toys, videos, or photos
		Match language and explanations to the child's understanding and family norms, tone, and perspective	Explore with the sibling the 3 W's—wonder, worries, and wishes
			Provide a distinction between the patient's situation and the sibling's well-being
			Match language and explanations to the child's understanding and family norms, tone, and perspective
Helping siblings to help the patient	Create connection and bonding opportunities	Anticipate and plan for potential impacts of sibling engagement	Anticipatory assessment of potential needs based on developmental level, temperament, and existing relationship
	Memory-making/building the story for/with the patient, sibling, and family.	Incorporate family expectations and preferences, norms, beliefs, and culture	Provide explanations of the patient's situation, their ability to interact, physical appearance and potential responses to sibling/interventions, an environment of care, the patient responses, parental responses, technology, personnel, and the activities in the PICU
	Prepare before, support during, and after all interactions with patients	Matching interventions to the sibling's preference for level, pace, and means of engagement	
		Protected time and resources for sibling support	Provide the child with tasks and activities, for example, journaling for the sibling, providing pictures/encouraging messages, holding a hand, and caring for a pet at home
		Ensure safety (emotional and physical) for both the patient and the visiting sibling	Provide photos or video for preparation to the patient and to the environment
		Secure healthcare team support for sibling presence/interactions	Explore options for presence at the bedside and/or alternatives, for example, video visits, sending notes, pictures, phone calls, and recorded messages
			If appropriate, allow time and space for private time between siblings with ready access to support if required
			Debrief and correct misconceptions/address fears after contact with the patient
Validation of sibling's and family's experience	Acknowledge sibling’s story and situate them within the “whole family” experience	Anticipatory and responsive support	Exploration of the sibling's perspective and understanding of their ill sibling’s situation
	Normalizing the sibling’s response, emotions, grief, concerns, and hopes	Support family members who are supporting the sibling(s)	Gentle correction or reframing of misconceptions
		Ensure flexibility and creative adaptation of plans to align with the changing situations	Abundant positive reinforcement of sibling's value and impact
			Partner and provide parents and family members with strategies for ongoing support
			Follow-up support at multiple time points during the hospitalization
			Provide peer resources where appropriate.
			Collaborate with the healthcare team to develop creative strategies to address important family events/mark milestones

### Proactive engagement and empowerment of sibling

Every family with a child in critical care should be assessed for child and youth family member needs. Parents might not be aware of their well child's experiences, or know of resources and expertise to support siblings, nor what benefits can be realized. However, parents can provide information about the family’s values and priorities and how to best support the sibling.

To engage siblings, opportunities for safe expression of emotions should be created. To facilitate emotional exploration with siblings, a safe environment is essential. Utilizing a private, child-friendly space is preferred when first meeting and assessing the sibling (23). Accessing parent and other family members’ perspectives on the child's temperament and coping strategies before meeting with the sibling establishes a foundation from which to begin building rapport. If parents and other family participate in conversations with the sibling, attention should be paid to preparing each participant for their role in that conversation. Preparation for these interactions can empower families to fulfill their role in supporting the patient and sibling(s) and will nurture existing family relationships. The extent to which an individual sibling is engaged in the patient's situation depends on the individual sibling's strengths, wishes, and the context of the illness.

### Acknowledge and honor relationships

Family connections can be under strain in times of critical illness experience. Acknowledging the connections between siblings helps integrate the well child into the family's story facilitating existing bonds (1). Connecting interventions intended to honor the relationship siblings share are matched to the sibling's and patient's emotional and cognitive capacity (24). Interventions acknowledging the sibling relationship can include the sibling's participation in family/ health care team discussions, being present with the patient, or designing alternative presence pathways. Supplementary Table S1 provides examples of interventions that assist with connection and acknowledging and maintaining sibling relationships.

### Helping siblings to help the patient

Assisting the sibling in engaging in the experience of the patient facilitates normalization and strengthens relationships (22). Interventions include soliciting the sibling’s input about the patient's likes and dislikes, active information sharing, spending time with the patient, designing and participating in special events or activities, and helping the patient directly with care and daily activities.

When collaborating with the sibling to help the patient, anticipatory assessment of the sibling's developmental level, temperament, and the existing relationship is essential. Interventions co-designed with the family and the sibling are preferred and these should be matched to the level of participation desired by the sibling. Examples of interventions include journaling for the sibling, providing pictures, sharing messages, selecting music to be played to the patient, or holding the patient's hand.

Options for the sibling to be at the bedside (and/or alternatives) should be explored. If visiting the patient is not possible, remote connections can be made through video visits, sending notes, pictures, phone calls, and recorded messages (25). If the sibling visits in person, thorough preparation for the sibling, family, and the critical care team is recommended. The sights, smells, and sounds of critical care and the plethora of professional care providers that the patient and their family interact with can be overwhelming. Support offered to siblings must prepare them for exposure to the critical care environment. Explanations of the setting, language, people, and equipment that the sibling may encounter are necessary. Siblings should also be prepared for the changed appearance, responses, and behavior of the patient. Viewing photos and videos of the patient and the care space prior to visiting can be helpful.

When facilitating interactions between the sibling and the patient consider exploration of the sibling's “wonders, worries, and wishes” (3 W's). Exploring the three W's prepares the child for their brother's or sister's changed appearance, behaviors, and ability to interact and can minimize any misinterpretation and fears (26). During bedside visits, it is advised that the sibling is provided with a purpose, task, or role to fulfill during the visit. Opportunities to connect siblings with the patient can be time-sensitive depending on the severity of the patient's illness. When possible, identifying and protecting time at the bedside for meaningful interactions between siblings should be considered. If appropriate, allow for a private time between the siblings with ready access to support if required. Create a plan for ending the visit should the situation change. It is essential to also debrief the visit to explore and correct any misconceptions or fears that arise after visiting (9, 27, 28). Helping siblings to help the patient requires the entire healthcare team's support. Frequent communication within the team and flexible, transparent planning are necessary to ensure a positive experience.

### Normalize the sibling's responses and the critical care experience for the family

Providing space and safety for the sibling to explore their feelings and open acknowledgment of these feelings as significant and valid provides a sense of normalcy in situations that feel overwhelming. Assisting siblings to understand why they are feeling the way they do helps reduce feelings of isolation and uncertainty. Actively normalizing the experience for siblings is ongoing as change is expected based on the patient's trajectory of illness recovery and the sibling's evolving understanding and engagement.

Educating and providing tools to adult family members to assist them in normalizing the sibling's emotions and concerns will assist in the anticipation of sibling needs and support timely responses as these needs arise. When both the critical care team and the family recognize, normalize, and validate the sibling's feelings the sibling can be an active participant and be present in the family's journey.

Facilitating non-medical activities of importance for the family can contribute to normalizing the experience. These activities can include observances, traditional celebrations, and family milestones. In times of critical childhood illness, the child and family's medical journey is often prioritized. By integrating non medical activities and events significant family occasions are not displaced (6). These events can occur alongside the medical plan of care and send a message of respect for family norms, cultural values, and wishes. Engaging sibling's input in decision-making and planning of family activities further consolidates their key role with their brother or sister. These connections will provide a meaningful opportunity for memory-making for the entire family (1).

## Operationalizing sibling support

Engaging with siblings is a continuous and ongoing process. Support for siblings requires the critical care team to recognize the importance of sibling well-being for the patient the sibling and the family. This can be hard to accomplish in the day-to-day activities of critical care. Activity is fast-paced and reactive to dynamic changes in clinical needs, resources, and organizational demands. Often the focus for critical care providers is the patient before them. Parents and adult families enjoy an established role with the team through their caregiving capacity, knowledge about their child, and as decision-makers for their child’s well-being. Siblings do not hold the same position within the day-to-day activities of critical care. They are often out of sight, and critical care providers must proactively reach out to understand how siblings might fit into the experience for the patient and family (2).

Unique factors such as family presence policy restricting children from the bedside and a physical environment that is not designed to accommodate children need to be identified and mitigated to engage siblings fully. If critical care team education is lacking the required knowledge and skills for sibling care will be limited, and the team's ability to recognize need and deliver appropriate interventions will be insufficient.

Uncertainty in terms of best practices and accountability for sibling care can also inhibit a team's ability to achieve the best sibling care (29). Some critical care units may have designated responsibility for sibling care to allied health roles such as social work or child life specialists (30). Sibling care interventions can be delivered by any knowledgeable critical care provider; however, success is best achieved when an overall culture of sibling inclusion is shared by the team and the processes of sibling care are grounded by organization policy.

Breaking sibling care into four pragmatic phases can assist the critical care team in operationalizing sibling support. These phases are (1) explore, (2) plan, (3) activate, and (4) follow through. The sibling support cycle is continuous. The phases of the cycle ground the continued assessment of the clinical context and individualized patient, sibling, and family needs throughout the critical care experience.

In the explore phase, determining if and to what extent the sibling would like to be connected to the patient is important. Options can be presented, and interventions adapted to fit the sibling's preference, capacity, and strengths.

In the plan phase, individualized and context-specific interventions are determined collaboratively with siblings and families. Selected interventions are then fit to the preferences, strengths, clinical context, and expectations of the child, sibling, and family.

The activate phase reflects the time when the sibling support plan is initiated and refined. Each sibling support intervention should be thoughtfully prepared, intentionally flexible in application, and include a purposeful evaluation of its effectiveness.

During the follow-through phase, the focus is on adapting and aligning interventions with the evolving goals for sibling support and modifying interventions in response to changes in the patient or the clinical situation. Attention to transparent communication and thoughtful determination of the next steps are the focus. There is also the opportunity to anticipate and plan for times when sibling needs can be predicted, for example, preparing for the transition to another area of care, changes in the patient's clinical situation (anticipated or unexpected), or changes in the family's situation. Essential in all phases of the cycle is the integration of child, sibling, and family perspectives and ensuring supportive relationships with the health care team.

## Conclusion

The critical childhood hospitalization of a brother, sister, or close child family member can disrupt family routines, and relationships and significantly impact the well child. Sibling well-being is essential to patient, sibling, and family adaptation. Providing adequate support for siblings has the potential to reduce family stress, elevate sibling and family well-being, and facilitate whole family adaptation to the patient's health situation (15).

Sibling support interventions need to fit the child, the family, and the illness factors. The impacts of individual interventions and the longer-term outcome for siblings of a critically ill child are not yet fully understood. Increasing critical care provider awareness of the sibling experience, exposing whole family outcomes, and evaluating interventions provided are important next steps in informing our collective understanding of, and ability to address these important needs. Developing mechanisms and skills to deliver consistent, strength-based sibling and family care is essential to facilitate whole family adaptation and support the best outcomes for patients and families.

## References

[1] Abela KM Casarez RL Kaplow J LoBiondo-Wood G. Siblings’ experience during pediatric intensive care hospitalization. J Pediatr Nurs. (2022) 64:111–8. 10.1016/j.pedn.2022.02.008 35287059

[2] Biswas M. Impact of critical illness on healthy siblings. Pediatr Nurs. (2022) 48(3):129–35. http://www.ajj.com/services/publication-services

[3] Gill MA. Making space for siblings in family-centered care. Pediatr Nurs. (2020) 46(1):48–51. https://www.proquest.com/scholarly-journals/making-space-siblings-family-centered-care/docview/2355333589/se-2

[4] Wincek JM. Promoting family-centered visitation makes a difference. AACN Clin Issues Crit Care Nurs. (1991) 2(2):293–8. 10.4037/15597768-1991-2015 2021514

[5] Hartling L Milne A Tjosvold L Wrightson D Gallivan J Newton AS. A systematic review of interventions to support siblings of children with chronic illness or disability. J Paediatr Child Health. (2014) 50(10):E26–38. 10.1111/j.1440-1754.2010.01771 20598075

[6] Rozdilsky JR. Enhancing sibling presence in pediatric ICU. Crit Care Nurs Clin North Am. (2005) 17(4):451–61. 10.1016/j.ccell.2005.07.001 16344214 PMC7118916

[7] Manning J Pinto N Rennick J Colville G Curley M. Conceptualizing post intensive care syndrome in children—the PICS-p framework. Pediatr Crit Care Med. (2018) 19(4):298–300. 10.1097/PCC.0000000000001476 29406379

[8] Keller BP Abney M Boles JC. Child life services for siblings of chronically ill children. J Child Life: Psycho Theory Prac. (2023) 4:2. 10.55591/001c.84322

[9] Blamires J Foster M Rasmussen S Zgambo M Mörelius E. The experiences and perceptions of healthy siblings of children with a long-term condition: umbrella review. J Pediatr Nurs. (2024) 77:191–203. 10.1016/j.pedn.2024.03.022 38574402

[10] Bichard E Butler AE. Visiting restrictions during COVID-19 pandemic—why are we still excluding siblings? Nurs Crit Care. (2023) 28(5):826–7. 10.1111/nicc.12898 36945768

[11] Abela KM Wardell D Rozmus C LoBiondo-Wood G. Impact of pediatric critical illness and injury on families: an updated systematic review. J Pediatr Nurs. (2020) 51:21–31. 10.1016/j.pedn.2019.10.013 31874458

[12] Rennick J. Reestablishing the parental role in a pediatric intensive care unit. J Pediatr Nurs. (1986) 1(1):40–4. PMID: . 3634805

[13] Colville G Darkins J Hesketh J Bennett V Alcock J Noyes J. The impact on parents of a child’s admission to intensive care: integration of qualitative findings from a cross-sectional study. Intensive Critical Care Nurse. (2009) 25(2):72–9. 10.1016/j.iccn.2008.10.002 19019677

[14] Macdonald ME Liben S Carnevale FA Cohen SR. An office or a bedroom? Challenges for family-centered care in the pediatric intensive care unit. J Child Health Care. (2012) 16(3):237–49. 10.1177/1367493511430678 22308544

[15] Thompson RH Bennett KL Snow ACW. Research in child life. In: Thompson RH, editor. The Handbook of Child Life: A Guide for Pediatric Psychosocial Care. Springfield, IL: Charles C Thomas Publisher, Ltd (2018). p. 55–103.

[16] Carnevale FA. Striving to recapture our previous life: the experience of families with critically ill children. Dynamics Official J Can Association Crit Care Nurs. (1999) 1(1):16–22. PMID: . 10347503

[17] Institute for Patient And Family-Centered Care. “About US” Institute For Patient And Family-Centered Care. (2024). Available online at: https://www.ipfcc.org/about/pfcc.html (Accessed June 8, 2024).

[18] Wright LM Leahey M. Calgary family intervention model: one way to think about change. J Marital Fam Ther. (1994) 20(4):381–95. 10.1111/j.1752-0606.1994.tb00128

[19] Foster M Whitehead L Maybee P. The parents, hospitalized child’s, and critical care providers perceptions and experiences of family-centered care within a pediatric critical care setting: a synthesis of quantitative research. J Fam Nurs. (2015) 22(1):6–73. 10.1177/1074840715618193 26706128

[20] Gottlieb LN. Strengths-Based Nursing Care: Health and Healing for Person and Family. New York, NY: Springer Publishing Co (2013).

[21] Tay J Widger K Stremler R. Self-reported experiences of siblings of children with life-threatening conditions: a scoping review. J Child Health Care. (2022) 26(4):517–30. 10.1177/13674935211026113 34116616 PMC9667075

[22] Romito B Jewell J Jackson M, AAP Committee on Hospital Care; Association of Child Life Professionals. Child life services. Pediatrics. (2021) 147(1):e2020040261. 10.1542/peds.2020-040261 33372119

[23] Beickert K Mora K. Transforming the pediatric experience: the story of child life. Pediatr Ann. (2017) 46(9):e345–51. 10.3928/19382359-20170810-01 28892551

[24] Eaton C. Sibling Support Strategies. Toronto: Internal Report, Hospital for Sick Children (2003).

[25] Bensink M Shergold J Lockwood L Little M Irving H Russell T Videophone support for an eight-year-old boy undergoing paediatric bone marrow transplantation. J Telemed Telecare. (2006) 12(5):266–8. 10.1258/135763306777889082 16848941

[26] Sick Kids. Talking to Kids about Serious Illness. The 6 C’s and the 3 W’s. (2023) Retrieved from: www.sickkids.ca

[27] Brauchle M Deffner T Nydahl P, ICU kids study group. Ten recommendations for child-friendly visiting policies in critical care. Intensive Care Med. (2023) 49(3):341–4. 10.1007/s00134-022-06974-w 36715706 PMC9998315

[28] Watson RS Choong K Colville G Crow S Dervan LA Hopkins RO Life after critical illness in children–toward an understanding of pediatric post-intensive care syndrome. J Pediatr. (2018) 198:16–24. 10.1016/j.jpeds.2017.12.084 29728304

[29] Olivier-D’Avignon M Dumont S Valois P Cohen SR. The needs of siblings of children with a life-threatening illness, part 1: conceptualization and development of a measure. Palliative and Supportive Care. (2017) 15(6):644–64. 10.1017/S1478951516 28122656

[30] Percelay JM Betts JM Chitkara MB Jewell JA Preuschoff CK Rauch DA. Child life services. Pediatrics. (2014) 133(5):e1471–8. 10.1542/peds.2014-0556 24777212

