# Supplementary material for: Supporting siblings during the critical illness hospitalization of a child: learning from experience
Source: Front Pediatr. 2024 Aug 26;12:1337491. doi: 10.3389/fped.2024.1337491 (PMC11381246; doi:10.3389/fped.2024.1337491)
Supplement: Supplementary file 1 [file Table1.pdf]

Supplementary Table 1: Interventions to support sibling connection

Goal addressed by the intervention	Intervention
**Demonstrating affection**	Hug and kisses crafts (for example, paper x's and o's to represent hugs and kisses to the patient)
	Fabric hugs
**Maintaining relationships**	Video or phone calls
	Recorded messages/stories, singing
	Letter writing
	Keeping a journal for/or with the patient
	Family play activities-games/shared craft activities
	Normalized play opportunities
	Connection stuffies
	Memory making (for example, handprints, picture frame decorating)
**Sibling involvement**	Art/decorations for the hospital room
	Selecting toys/books for the patient
	Choosing photos to send to patient
	Given a job at home – taking care of patient's belongings/pet
	Contribute to celebrations and milestone planning
	Inclusion in decision making process
**Addressing anxiety**	Expressive play (for example, journalling, letter writing, drawing)
	Provide choice (or example, length/time of visit)
	Protected time for visit-limit medical activities in that interval
	Staged approaches to visiting- (photos of patient and bedside)
	De-medicalize the environment where possible
	Establish check in times for follow-up
	Referrals to mental health and wellness resources as needed

Maintenance of sibling connections can be achieved through actions in 4 main areas: 1] demonstrating mutual affection, 2] maintaining sibling relationships, 3] actively engaging the sibling in the patient's care, and 4] mitigating sibling anxiety. Interventions that can support in these areas are listed here. All interventions can be adapted and fit to the clinical context, development stage and the preferences and priorities of the child and family.
